# 3-[Chloro­(phen­yl)meth­yl]-6-methyl-1,2-benzoxazole

**DOI:** 10.1107/S1600536811042462

**Published:** 2011-10-22

**Authors:** M. Kayalvizhi, G. Vasuki, K. Ramamurthi, A. Veerareddy, G. Laxminarasimha

**Affiliations:** a Department of Physics, Kunthavai Naachiar Govt. Arts College(W)(Autonomous), Thanjavur 7, India; b Crystal Growth and Thin Film Laboratory, School of Physics, Bharathidasan University, Tiruchirappalli 24, India; c R & D Laboratories, Suven Life Sciences Limited, Hyderabad 55, Andhra Pradesh, India

## Abstract

The title compound, C_15_H_12_ClNO, is a functionalized 1,2-benzoxazole with a chloro­(phen­yl)methyl substituent. The mol­ecule is V-shaped, the dihedral angle between the mean plane of the 1,2-benzoxazole system [maximum deviation = 0.023 (3) Å for the N atom] and the phenyl ring being 70.33 (14)°. There are no hydrogen-bonding inter­actions in the crystal structure, which is stabilized by van der Waals inter­actions only.

## Related literature

For the synthesis of the title compound, see: Veerareddy *et al.* (2011 ▶). For related structures, see: Atovmyan & Aliev (1994 ▶); Hu *et al.* (2009 ▶); Korlyukov *et al.* (2003 ▶).
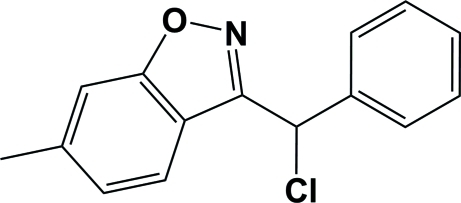


## Experimental

### 

#### Crystal data

C_15_H_12_ClNO
*M* *_r_* = 257.71
Monoclinic, 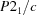

*a* = 13.2075 (8) Å
*b* = 6.5888 (4) Å
*c* = 15.1224 (8) Å
β = 103.738 (3)°
*V* = 1278.33 (13) Å^3^
*Z* = 4
Mo *K*α radiation
μ = 0.29 mm^−1^
*T* = 293 K
0.30 × 0.30 × 0.20 mm

#### Data collection

Bruker Kappa APEXII CCD diffractometer
Absorption correction: multi-scan (*SADABS*; Bruker, 2001 ▶) *T*_min_ = 0.919, *T*_max_ = 0.945
10507 measured reflections
2087 independent reflections
1621 reflections with *I* > 2σ(*I*)
*R*_int_ = 0.028

#### Refinement

*R*[*F* ^2^ > 2σ(*F* ^2^)] = 0.041
*wR*(*F* ^2^) = 0.125
*S* = 1.06
2087 reflections
163 parameters
H-atom parameters constrained
Δρ_max_ = 0.32 e Å^−3^
Δρ_min_ = −0.28 e Å^−3^

### 

Data collection: *APEX2* (Bruker, 2004 ▶); cell refinement: *APEX2* and *SAINT* (Bruker, 2004 ▶); data reduction: *SAINT* and *XPREP* (Bruker, 2004 ▶); program(s) used to solve structure: *SHELXS97* (Sheldrick, 2008 ▶); program(s) used to refine structure: *SHELXL97* (Sheldrick, 2008 ▶); molecular graphics: *ORTEP-3* (Farrugia, 1997 ▶) and *Mercury* (Macrae *et al.*, 2008) ▶; software used to prepare material for publication: *PLATON* (Spek, 2009 ▶).

## Supplementary Material

Crystal structure: contains datablock(s) I, global. DOI: 10.1107/S1600536811042462/su2326sup1.cif

Structure factors: contains datablock(s) I. DOI: 10.1107/S1600536811042462/su2326Isup2.hkl

Supplementary material file. DOI: 10.1107/S1600536811042462/su2326Isup3.cml

Additional supplementary materials:  crystallographic information; 3D view; checkCIF report

## Figures and Tables

**Fig. 1.**
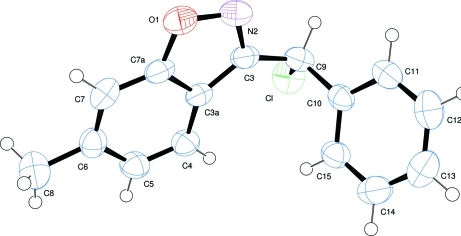
The molecular structure of compound (I), showing the numbering sheme and displacement ellipsoids drawn at the 50% probability level.

**Fig. 2.**
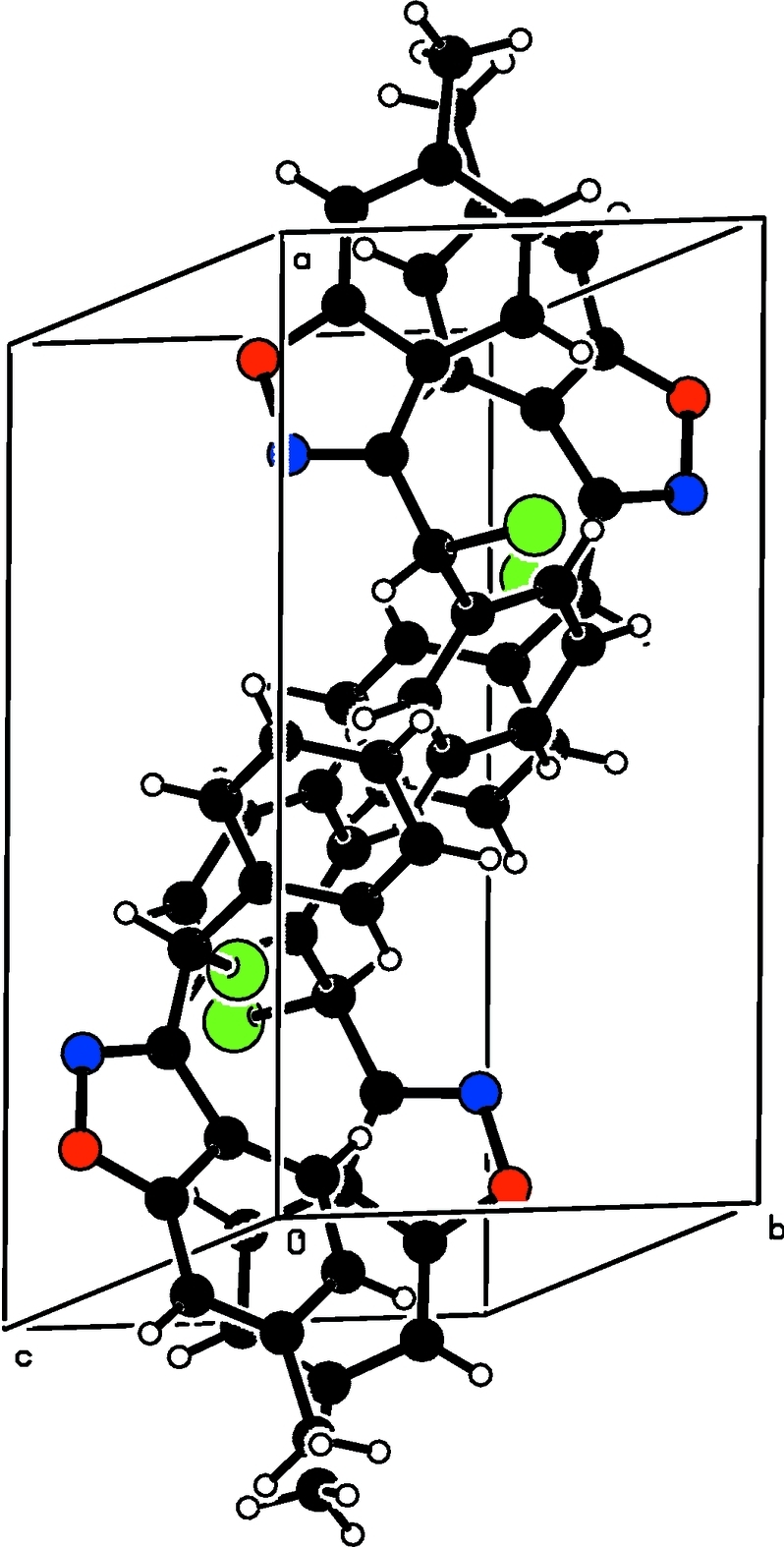
A view of the crystal packing of compound (I).

## supplementary crystallographic information

### Comment

Benzisoxazole is an aromatic organic compound with a molecular formula C_7_H_5_NO containing a benzene-fused isoxazole ring structure. Benzisoxazole is primarily used in industry and research. Being a heterocyclic compound, benzisoxazole finds use in research as a starting material for the synthesis of larger, usually bioactive structures. Isoxazole and benzisoxazole are important classes of nitrogen-oxygen containing heterocycles. They have extensive applications as structural units of various biologically important molecules and as useful intermediates in medicinal chemistry. Among them, 3–substituted–1,2–benzisoxazole and their derivatives are emerging as potential antipsychotic compounds. For example, 1,2–benzisoxazole–3–methanesulfonamide, also known as zonisamide, is an efficient antiseizure agent. It has been reported that it blocks the repetitive firing of voltage–sensitive sodium channels and reduces voltage–sensitive T–type calcium currents (Veerareddy *et al.*, 2011).

In molecular structure of the title functionalized 1,2-benzoxazole compound, (I), is illustrated in Fig. 1. The bond length and angles are in agreement with those found for closely related structures, for example, 6-tert-butyl-4,5-dichloro-3-ethyl-4,5-dihydro-2,1-benzoisoxazole (II) [Atovmyan & Aliev, 1994], 3-(1,3-dioxolan-2-yl)-4,6-dinitrobenzo[d]isoxazole (III) [Korlyukov *et al.*, 2003], and N-Phenyl-4-(8-phenyl-4,5-dihydro-1,2-benzoxazolo- [4,5-d]thiazol-2-yl)-piperidine-1-carboxamide (IV) [Hu *et al.*, 2009]. The widening of the exocyclic angle C10—C9—C3 [113.4 (2)°] from the normal value of 109° may be due to repulsion between neighbouring H atoms [H9··· H11 = 2.2495 (1) Å]. The exocyclic angles C9—C3—C3a [132.1 (2)°] and C3—C3a—C4 [138.2 (2)°] deviate significantly from the normal value of 120° and this may be due to the intramolecular non-bonded interactions between the chlorine atom and H-atom H4 at C10 [Cl··· H4 = 3.1169 (7) Å]. The isoxazole ring (O1,N2,C3,C3a,C7a) is planar [max. deviation 0.007 (3) Å], with the chloro(phenyl)methyl substituent being nearly normal to the plane of the five membered ring [N2-C3-C9-C10 = 100.1 (3)°], similar to the situation in compound (II) (Atovmyan & Aliev, 1994). The dihedral angle between the mean plane of the 1,2-benzoxazole [max. deviation 0.023 (3) Å] and the phenyl ring (C10-C15) is 70.33 (14)°.

Crystal packing of compound (I) is illustrated in Fig. 2. There are no significant non-bonded interactions present and the crystal structure is stabilized by van der Waals interactions only.

### Experimental

The compound was synthesized following the published procedure (Veerareddy *et al.*, 2011).

### Refinement

All the H atoms were positioned geometrically and treated as riding on their parent atoms, with C—H = 0.93Å (aromatic), 0.98Å (methine) and 0.96Å (methyl), and refined using a riding model with *U*_iso_(H )= 1.2*U*_eq_(C) or 1.5*U*_eq_(methyl C).

### Crystal data

**Table:**

C_15_H_12_ClNO	*F*(000) = 536
*M**_r_* = 257.71	*D*_x_ = 1.339 Mg m^−^^3^
Monoclinic, *P*2_1_/*c*	Mo *K*α radiation, λ = 0.71073 Å
Hall symbol: -P 2ybc	Cell parameters from 25 reflections
*a* = 13.2075 (8) Å	θ = 20–30°
*b* = 6.5888 (4) Å	µ = 0.29 mm^−^^1^
*c* = 15.1224 (8) Å	*T* = 293 K
β = 103.738 (3)°	Block, colourless
*V* = 1278.33 (13) Å^3^	0.30 × 0.30 × 0.20 mm
*Z* = 4	

### Data collection

**Table:**

Bruker Kappa APEXII CCD diffractometer	2087 independent reflections
Radiation source: fine-focus sealed tube	1621 reflections with *I* > 2σ(*I*)
graphite	*R*_int_ = 0.028
ω and φ scan	θ_max_ = 24.4°, θ_min_ = 2.8°
Absorption correction: multi-scan (*SADABS*; Bruker, 2001)	*h* = −15→15
*T*_min_ = 0.919, *T*_max_ = 0.945	*k* = −5→7
10507 measured reflections	*l* = −17→17

### Refinement

**Table:**

Refinement on *F*^2^	Primary atom site location: structure-invariant direct methods
Least-squares matrix: full	Secondary atom site location: difference Fourier map
*R*[*F*^2^ > 2σ(*F*^2^)] = 0.041	Hydrogen site location: inferred from neighbouring sites
*wR*(*F*^2^) = 0.125	H-atom parameters constrained
*S* = 1.06	*w* = 1/[σ^2^(*F*_o_^2^) + (0.0577*P*)^2^ + 0.7285*P*] where *P* = (*F*_o_^2^ + 2*F*_c_^2^)/3
2087 reflections	(Δ/σ)_max_ < 0.001
163 parameters	Δρ_max_ = 0.32 e Å^−^^3^
0 restraints	Δρ_min_ = −0.28 e Å^−^^3^

### Special details

**Table:**

Geometry. All e.s.d.'s (except the e.s.d. in the dihedral angle between two l.s. planes) are estimated using the full covariance matrix. The cell e.s.d.'s are taken into account individually in the estimation of e.s.d.'s in distances, angles and torsion angles; correlations between e.s.d.'s in cell parameters are only used when they are defined by crystal symmetry. An approximate (isotropic) treatment of cell e.s.d.'s is used for estimating e.s.d.'s involving l.s. planes.
Refinement. Refinement of *F*^2^ against ALL reflections. The weighted *R*-factor *wR* and goodness of fit *S* are based on *F*^2^, conventional *R*-factors *R* are based on *F*, with *F* set to zero for negative *F*^2^. The threshold expression of *F*^2^ > σ(*F*^2^) is used only for calculating *R*-factors(gt) *etc*. and is not relevant to the choice of reflections for refinement. *R*-factors based on *F*^2^ are statistically about twice as large as those based on *F*, and *R*- factors based on ALL data will be even larger.

### Fractional atomic coordinates and isotropic or equivalent isotropic displacement parameters (Å^2^)

**Table:**

	*x*	*y*	*z*	*U*_iso_*/*U*_eq_	
C14	0.3858 (2)	0.3538 (4)	0.10340 (19)	0.0625 (7)	
H14	0.3663	0.4825	0.0803	0.075*	
C15	0.33247 (19)	0.2630 (4)	0.16026 (17)	0.0514 (6)	
H15	0.2771	0.3303	0.1756	0.062*	
C10	0.36083 (17)	0.0718 (3)	0.19482 (15)	0.0426 (6)	
C11	0.44364 (18)	−0.0247 (4)	0.17192 (16)	0.0512 (6)	
H11	0.4639	−0.1530	0.1953	0.061*	
C12	0.4965 (2)	0.0676 (5)	0.11477 (18)	0.0614 (7)	
H12	0.5521	0.0012	0.0995	0.074*	
C13	0.4678 (2)	0.2562 (5)	0.08037 (18)	0.0637 (8)	
H13	0.5035	0.3181	0.0416	0.076*	
C9	0.30211 (18)	−0.0408 (4)	0.25392 (16)	0.0499 (6)	
H9	0.3434	−0.1605	0.2783	0.060*	
C3	0.19728 (18)	−0.1135 (3)	0.20317 (16)	0.0453 (6)	
C3A	0.10029 (17)	−0.0106 (3)	0.17045 (15)	0.0407 (5)	
C4	0.05889 (18)	0.1827 (4)	0.17530 (16)	0.0474 (6)	
H4	0.0998	0.2874	0.2060	0.057*	
C5	−0.04334 (19)	0.2133 (4)	0.13360 (17)	0.0512 (6)	
H5	−0.0716	0.3417	0.1366	0.061*	
C6	−0.10804 (18)	0.0600 (4)	0.08627 (16)	0.0486 (6)	
C7	−0.06762 (19)	−0.1303 (4)	0.08068 (17)	0.0526 (6)	
H7	−0.1082	−0.2348	0.0494	0.063*	
C7A	0.03587 (19)	−0.1596 (3)	0.12364 (17)	0.0476 (6)	
C8	−0.2204 (2)	0.1048 (5)	0.0429 (2)	0.0708 (8)	
H8A	−0.2352	0.2441	0.0536	0.106*	
H8B	−0.2642	0.0181	0.0687	0.106*	
H8C	−0.2336	0.0809	−0.0215	0.106*	
N2	0.19137 (18)	−0.3037 (3)	0.17984 (18)	0.0657 (6)	
O1	0.08903 (15)	−0.3377 (3)	0.12767 (15)	0.0693 (6)	
Cl	0.28938 (6)	0.11252 (13)	0.34931 (5)	0.0704 (3)	

### Atomic displacement parameters (Å^2^)

**Table:**

	*U*^11^	*U*^22^	*U*^33^	*U*^12^	*U*^13^	*U*^23^
C14	0.0699 (18)	0.0508 (17)	0.0611 (16)	−0.0066 (14)	0.0044 (14)	0.0094 (13)
C15	0.0511 (14)	0.0432 (15)	0.0587 (15)	0.0055 (11)	0.0106 (11)	0.0002 (12)
C10	0.0392 (12)	0.0423 (14)	0.0416 (12)	0.0019 (10)	0.0006 (9)	−0.0024 (10)
C11	0.0486 (14)	0.0475 (15)	0.0531 (14)	0.0081 (12)	0.0031 (11)	−0.0021 (12)
C12	0.0488 (15)	0.079 (2)	0.0567 (16)	0.0005 (14)	0.0144 (12)	−0.0122 (15)
C13	0.0619 (17)	0.078 (2)	0.0519 (15)	−0.0193 (15)	0.0139 (13)	0.0000 (15)
C9	0.0551 (15)	0.0430 (14)	0.0513 (14)	0.0105 (11)	0.0120 (11)	0.0057 (11)
C3	0.0534 (14)	0.0350 (13)	0.0504 (13)	0.0023 (10)	0.0179 (11)	0.0070 (10)
C3A	0.0484 (13)	0.0326 (12)	0.0441 (12)	−0.0027 (10)	0.0169 (10)	0.0027 (10)
C4	0.0526 (14)	0.0376 (14)	0.0511 (14)	−0.0020 (11)	0.0103 (11)	−0.0060 (11)
C5	0.0556 (15)	0.0424 (15)	0.0569 (14)	0.0056 (12)	0.0158 (12)	−0.0026 (12)
C6	0.0458 (13)	0.0564 (17)	0.0450 (13)	−0.0035 (12)	0.0137 (10)	0.0013 (11)
C7	0.0540 (15)	0.0498 (16)	0.0560 (15)	−0.0153 (12)	0.0173 (12)	−0.0054 (12)
C7A	0.0579 (15)	0.0313 (13)	0.0572 (14)	−0.0044 (11)	0.0207 (12)	0.0029 (11)
C8	0.0540 (16)	0.080 (2)	0.0760 (19)	−0.0010 (14)	0.0105 (14)	−0.0066 (16)
N2	0.0642 (15)	0.0356 (13)	0.0970 (18)	0.0053 (10)	0.0186 (13)	0.0041 (12)
O1	0.0672 (12)	0.0324 (10)	0.1063 (16)	−0.0036 (9)	0.0168 (11)	−0.0070 (10)
Cl	0.0770 (5)	0.0849 (6)	0.0506 (4)	−0.0038 (4)	0.0177 (3)	−0.0093 (3)

### Geometric parameters (Å, °)

**Table:**

C14—C15	1.371 (4)	C3—C3A	1.430 (3)
C14—C13	1.374 (4)	C3A—C7A	1.380 (3)
C14—H14	0.9300	C3A—C4	1.395 (3)
C15—C10	1.381 (3)	C4—C5	1.363 (3)
C15—H15	0.9300	C4—H4	0.9300
C10—C11	1.378 (3)	C5—C6	1.404 (4)
C10—C9	1.510 (3)	C5—H5	0.9300
C11—C12	1.375 (4)	C6—C7	1.374 (4)
C11—H11	0.9300	C6—C8	1.502 (4)
C12—C13	1.365 (4)	C7—C7A	1.380 (3)
C12—H12	0.9300	C7—H7	0.9300
C13—H13	0.9300	C7A—O1	1.361 (3)
C9—C3	1.494 (3)	C8—H8A	0.9600
C9—Cl	1.801 (2)	C8—H8B	0.9600
C9—H9	0.9800	C8—H8C	0.9600
C3—N2	1.299 (3)	N2—O1	1.412 (3)
			
C15—C14—C13	120.5 (3)	C7A—C3A—C4	118.4 (2)
C15—C14—H14	119.7	C7A—C3A—C3	103.5 (2)
C13—C14—H14	119.7	C4—C3A—C3	138.2 (2)
C14—C15—C10	120.1 (2)	C5—C4—C3A	118.0 (2)
C14—C15—H15	119.9	C5—C4—H4	121.0
C10—C15—H15	119.9	C3A—C4—H4	121.0
C11—C10—C15	119.1 (2)	C4—C5—C6	123.0 (2)
C11—C10—C9	118.2 (2)	C4—C5—H5	118.5
C15—C10—C9	122.7 (2)	C6—C5—H5	118.5
C12—C11—C10	120.3 (3)	C7—C6—C5	119.3 (2)
C12—C11—H11	119.8	C7—C6—C8	120.7 (2)
C10—C11—H11	119.8	C5—C6—C8	120.0 (2)
C13—C12—C11	120.4 (2)	C6—C7—C7A	117.0 (2)
C13—C12—H12	119.8	C6—C7—H7	121.5
C11—C12—H12	119.8	C7A—C7—H7	121.5
C12—C13—C14	119.6 (3)	O1—C7A—C3A	110.0 (2)
C12—C13—H13	120.2	O1—C7A—C7	125.8 (2)
C14—C13—H13	120.2	C3A—C7A—C7	124.3 (2)
C3—C9—C10	113.4 (2)	C6—C8—H8A	109.5
C3—C9—Cl	109.88 (16)	C6—C8—H8B	109.5
C10—C9—Cl	110.95 (17)	H8A—C8—H8B	109.5
C3—C9—H9	107.5	C6—C8—H8C	109.5
C10—C9—H9	107.5	H8A—C8—H8C	109.5
Cl—C9—H9	107.5	H8B—C8—H8C	109.5
N2—C3—C3A	111.9 (2)	C3—N2—O1	107.0 (2)
N2—C3—C9	116.0 (2)	C7A—O1—N2	107.68 (18)
C3A—C3—C9	132.1 (2)		
			
C13—C14—C15—C10	−0.1 (4)	C9—C3—C3A—C4	−5.6 (5)
C14—C15—C10—C11	0.5 (4)	C7A—C3A—C4—C5	0.2 (3)
C14—C15—C10—C9	−176.9 (2)	C3—C3A—C4—C5	−178.3 (2)
C15—C10—C11—C12	−0.6 (3)	C3A—C4—C5—C6	−0.1 (4)
C9—C10—C11—C12	176.9 (2)	C4—C5—C6—C7	−0.3 (4)
C10—C11—C12—C13	0.3 (4)	C4—C5—C6—C8	179.3 (2)
C11—C12—C13—C14	0.2 (4)	C5—C6—C7—C7A	0.6 (3)
C15—C14—C13—C12	−0.3 (4)	C8—C6—C7—C7A	−179.0 (2)
C11—C10—C9—C3	−106.5 (2)	C4—C3A—C7A—O1	−178.9 (2)
C15—C10—C9—C3	71.0 (3)	C3—C3A—C7A—O1	0.1 (3)
C11—C10—C9—Cl	129.31 (19)	C4—C3A—C7A—C7	0.2 (4)
C15—C10—C9—Cl	−53.2 (3)	C3—C3A—C7A—C7	179.1 (2)
C10—C9—C3—N2	100.1 (3)	C6—C7—C7A—O1	178.3 (2)
Cl—C9—C3—N2	−135.1 (2)	C6—C7—C7A—C3A	−0.6 (4)
C10—C9—C3—C3A	−76.4 (3)	C3A—C3—N2—O1	1.3 (3)
Cl—C9—C3—C3A	48.4 (3)	C9—C3—N2—O1	−175.9 (2)
N2—C3—C3A—C7A	−0.9 (3)	C3A—C7A—O1—N2	0.7 (3)
C9—C3—C3A—C7A	175.7 (2)	C7—C7A—O1—N2	−178.4 (2)
N2—C3—C3A—C4	177.7 (3)	C3—N2—O1—C7A	−1.2 (3)
